# Tissue-Specific Content of Polyunsaturated Fatty Acids in (n-3) Deficiency State of Rats

**DOI:** 10.3390/foods11020208

**Published:** 2022-01-12

**Authors:** Amruta Kulkarni, Ai Zhao, Baoru Yang, Yumei Zhang, Kaisa M. Linderborg

**Affiliations:** 1 Food Chemistry and Food Development, Department of Life Technologies, University of Turku, 20520 Turku, Finland; amruta.kulkarni@utu.fi (A.K.); bayang@utu.fi (B.Y.); 2 Vanke School of Public Health, Tsinghua University, Beijing 100083, China; xiaochaai@163.com; 3 Department of Nutrition & Food Hygiene, School of Public Health, Peking University Health Science Center, Beijing 100191, China

**Keywords:** docosahexaenoic acid, (n-3) polyunsaturated fatty acids, (n-3) deficiency, α-linolenic acid, lipid metabolism, fatty acid composition

## Abstract

The dietary intake of fatty acids (FAs) affects the composition and distribution of FAs in the body. Here, a first-generation (n-3)-deficiency study was conducted by keeping young (age 21 ± 2 days) Sprague–Dawley male rats on a peanut-oil-based diet for 33 days after weaning in order to compare the effect of mild (n-3)-deficiency on the lipid composition of different organs and feces. Soybean-oil-based diet was used as a control. The plasma FA levels corresponded to FAs levels in the organs. Lower docosahexaenoic acid (DHA) content was detected in the plasma, brain, testis, visceral fat, heart, and lungs of the (n-3)-deficient group, whereas the DHA content of the eye and feces did not differ between the experimental groups. The DHA content of the brains of the (n-3)-deficient group was 86% of the DHA content of the brains of the (n-3)-adequate group. The DHA level of the organs was affected in the order of visceral fat > liver triacylglycerols > lung > heart > liver phospholipids > testis > eye > brain, with brain being least affected. The low levels of (n-3) FAs in the liver, brain, eye, heart, and lung were offset by an increase in the (n-6) FAs, mainly arachidonic acid. These results indicate that, in rats, adequate maternal nutrition during pregnancy and weaning does not provide enough (n-3) FAs for 33 days of an (n-3)-deficient diet. Results of this study can be used also to evaluate the conditions needed to reach mild (n-3) deficiency in the first generation of rats and to evaluate the feasibility to collect data from a variety of organs or only selected ones.

## 1. Introduction

The dietary intake of fatty acids (FAs) affects the overall composition and distribution of FAs in the body. Studies from the 1920s by Burr and Burr [1] (published in 1973) indicated even then that lack of dietary fat leads to alterations in development and metabolism, resulting in disease and even death at an early age. Mammals, including humans, lack the necessary enzymes (Δ-12 and Δ-15 desaturases) to synthesize linoleic (18:2(n-6), LA) and α-linolenic (18:3(n-3), ALA) acids, and thus, they are considered essential FAs that must originate from the diet. In the body, ALA and LA are precursors of long-chain (n-3) and (n-6) polyunsaturated FAs (LC PUFAs) of the same omega family, most importantly arachidonic acid (20:4(n-6), ARA), eicosapentaenoic acid (20:5(n-3), EPA), and docosahexaenoic acid (22:6(n-3), DHA). The biosynthesis pathway of LC PUFAs from ALA and LA involves a shared enzymatic cascade, including the rate-limiting step of Δ6 desaturation, and thus, there is a competition for enzyme activities between (n-3) and (n-6) families of FAs, as shown in early studies of rat liver microsomes [2]. Thus, not only the total intake but also the dietary balance between PUFAs of the different omega families affect their metabolism [3,4,5].

Typically, plant seed oils serve as sources for both LA and ALA but in different concentrations and ratios, whereas animal fat is typically a poor source of especially ALA. Nuts are typically short of ALA, and peanut oil is known to practically lack (n-3) FAs [6]. Seafood is an excellent source of (n-3) PUFAs, but its consumption varies greatly among regions and cultures and is generally lower than recommended. Based on the global consumption of key dietary fats and oils in 2010, it is estimated that less than 20% of the world’s population receives (n-3) PUFAs from seafood in quantities equal to or more than 250 mg/day [7]. In the dietary shift towards more solid fats and more animal based foods, the (n-6)/(n-3) ratio of PUFAs in the human diet has been increasing dramatically, and multiple studies indicate a low (n-3) PUFAs status in humans [8,9].

The deficiency of LC (n-3) PUFAs leads to the pathogenesis of several diseases, such as neurodegenerative diseases, cancer, depression, and inflammatory and autoimmune diseases, whereas the supplementation of LC (n-3) PUFAs, such as DHA and EPA, exerts a suppressive effect on these health problems [10,11,12] and maintains optimal health [13,14].

Physiological consequences of (n-3) deficiency have been previously studied in rodents, guinea pigs, and non-human primates. In rodents, (n-3) PUFA deprivation has resulted in a decrease of DHA levels in the brain [15], a compensatory increase of (n-6) PUFAs in the brain [16], and upregulation of the conversion of the circulating ALA to DHA in the liver but not in the brain [17]. Elevated anxiety, aggression, increased immobility time, and vulnerability to stress have also been reported in rodents fed a low-(n-3) PUFA diet [18]. In guinea pigs, (n-3) deficiency has resulted in decreased DHA in the phospholipids of the retina [19,20,21], brain, liver, and heart [19] as well as significant reductions in the amplitudes of receptoral and postreceptoral subcomponents and in the sensitivity of the receptoral subcomponent of the electroretinogram [22]. In rhesus monkeys, restriction of LC (n-3) PUFAs during the prenatal period affected the FA compositions of retinal [23] and neural membranes [24], retinal and visual functions [23,24], as well as the behavior of the animals [25].

Analyses of the composition of tissues subjected to the (n-3) deficiency have most often included the brain, retina, liver, and plasma [15,26,27,28,29], while very few previous investigations are published for other organs, such as the heart, lung, and testis. One study from the late 1970s included analysis of the fatty acid compositions of liver, brain, kidney, spleen, heart, muscle, gastrointestinal tract, lung, ovary, testis, adrenal, plasma, erythrocytes, retina, and adipose tissue in a study of (n-3) deficiency, but the male and female rats studied were from the second generation of deficient rats [30]. Previous studies showed a decline in DHA level of heart, testis, brain and, liver [31]; heart, lung, testis, brain cells, retina, muscle, liver, and kidney [32]; and lung, testis, brain cells, kidney, retina, and liver [33] in rats of third or fourth generations fed with (n-3)-deficient diet as compared to (n-3)-adequate diet. A few studies with the first generation of (n-3)-deficient rats have included the heart and liver [34]; brain [15]; brain, liver heart, and plasma [27]; liver and plasma [35]; and brain [26], but they did not include several organs in the same study to compare the tissue specific response of (n-3) deficiency. Thus, previous information available on the extent of the effect of (n-3) deficiency on the rat tissues from the first generation is scarce. The present study compares the effect caused by diets containing either soybean oil or peanut oil in the first generation of (n-3)-deficient rats. Animal studies in the first generation of (n-3) deficiency are developed to evaluate the impact of an essential nutrient-deficient diet post-weaning in offspring born to dams who are maintained on essential nutrient adequate diet throughout pregnancy. However, achieving a severe deficiency is difficult, as LC (n-3) PUFA, particularly DHA, originating from the perinatal period and mother’s milk during the suckling period, is efficiently maintained in the brain. Thus, second-generation studies have also been carried out in offspring born to dams kept on the (n-3)-deficient diet throughout their post-weaning lives. Even third-generation deficiency studies are possible [32,36,37], but conducting such second- and third-generation studies may be limited by time and resources, and they rarely can be extrapolated to real-life human situations.

Here, a first-generation (n-3)-deficiency study was conducted in rats in order to compare the effect of mild (n-3) deficiency on the lipid composition of different organs. Mild deficiency was chosen instead of a second-generation deficiency study, as a (n-3) deficiency with clear clinical symptoms is uncommon in humans. This study focuses on the lipid compositions of a variety of tissues, including the brain, eyes, liver, visceral fat, testis, heart, and lungs as well as plasma and feces in order to evaluate the tissue-specific impact of (n-3)-deficiency status, previously analyzed only from severe (n-3)-deficiency states. To our knowledge, the effect of (n-3) deficiency on rat feces had never been studied previously. The deficiency state was induced by keeping young male rats on a peanut-oil-based diet for 33 days in order to study the deficiency state in young adult animals. This research answers questions on (1) whether a relatively short-term, first-generation (n-3) deficiency at the young adult age can be seen in the FA composition of different organs and (2) whether the composition, mainly ratios between ARA, EPA, and DHA, of certain organs, affected more than those of others.

## 2. Materials and Methods

### 2.1. Ethical Concerns

The animal experiment was conducted following the protocol approved by the Medical Ethics Research Board of the Peking University Health Science Center, China (Study identifier LA2016043).

### 2.2. Diets, Animal Trial, and Sample Collection

Two rodent AIN-93G diets were prepared using either soybean oil ((n-3)-adequate diet) or peanut oil ((n-3)-deficient diet). Diets were matched for the non-fat nutrients (Table 1). The fatty acid composition of the oils used in the feed is shown in Table 2. The (n-3)-adequate diet group was also included as a control (referred to as normal feed group) in our earlier study evaluating the bioavailability of DHA from different stereospecific positions of triacylglycerols (TAGs) [38].

The dams were fed with soybean-oil-based feed. Later, young male Sprague–Dawley rats (age 21 ± 2 days) were kept for 7 days on adaptive feeding with standard AIN-93G feed containing soybean oil as a source of (n-3) fatty acids. Then, rats were randomly divided into two groups: (n-3)-adequate group and (n-3)-deficient group (*n* = 12 in each group). One rat from the (n-3)-deficient group died during the induction phase and thus did not receive the intervention. The animal trial model is shown in Supplementary Figure S1. Feeding of (n-3)-adequate group was continued with the soybean oil containing AIN-93G diet, and the (n-3)-deficient group was kept on the peanut oil containing AIN-93G diet with a low-(n-3) FA diet for the following 33 days. During the first four weeks, 4 rats were housed in one cage, and thereafter, rats were housed individually for the remaining 5 days. Rats were fed *ad libitum* throughout the experiment. The weights of the rats at the end of the experiment are shown in Table 3.

Feces were collected as a pooled sample from the last 5 days as described earlier [38]. The fecal samples were weighed and stored at −80 °C. The rats were sedated by inhaling isoflurane and sacrificed in fasting state with exsanguination. Blood samples were collected in the dry tubes without anticoagulant after the femoral artery was cut off and then centrifuged. Plasma was collected and stored at −80 °C. For this study, the liver, brain, eyes, testis, visceral fat, lung, and heart were collected and analyzed. All the organs from each rat were weighed and thereafter stored at −80 °C until analysis.

### 2.3. Lipid Extraction, Fractionation into Neutral and Polar Rich Lipids, and Methylation of FAs

Lipids were extracted from the plasma, feces, liver, brain, eye, testis, visceral fat, lung, and heart, and their lipid content and fatty acid composition were analyzed. The liver, eye, testis, visceral fat, lung, and heart were diced into pieces on an ice bath, and pieces were randomly selected for the lipid extraction. Brains were weighed, frozen in liquid nitrogen, and homogenized using Bio-Gen PRO200 Homogenizer (PRO Scientific, Monroe, CT, USA). The homogenate was divided into two portions, and one portion was stored at −80 °C for lipid analysis. The tissue of the liver, eye, testis, visceral fat, lung, heart, and brain were weighed, followed by homogenization in methanol (10 mL of MeOH for 1 g of tissue) with an Ultra-Turrax T 25 instrument (IKA Werke GmbH & Co. KG, Staufen, Germany) in four intervals for 30 s at 8000 rpm. The homogenized tissue in methanol was stored at −80 °C.

Analyses were carried out in duplicate for all the organs except the eye. Total lipids were extracted with a modified Folch method using HPLC grade methanol, chloroform, and 0.88% potassium chloride (KCl) in milli-Q water [39,40] from plasma and feces (≈200 mg) as well as from homogenate of the liver, visceral fat, testis, brain, heart, and lung (each aliquot ≈200 mg of tissue for the brain; ≈500 mg of tissue for other organs) and one eyeball of each rat (≈106–138 mg). Triheptadecanoin (Larodan Fine Chemicals AB, Malmö, Sweden) was added as an internal standard to the samples of liver, brain, eye, visceral fat, testis, lung, and heart tissue aliquot as well as to the plasma and fecal samples and dinonadecanoylphosphatidylcholine (Larodan Fine Chemicals AB, Malmö, Sweden) to aliquots of the plasma and the liver, testis, lung, and heart tissues.

As plasma and liver were assumed to contain significant amounts of both neutral and polar lipids, they were analyzed after fractionation into a neutral lipid-rich fraction (TAG fraction) and a polar lipid-rich fraction (PL fraction) with solid phase extraction using Sep-Pak Vac 1cc silica cartridges (Waters, Dublin, Ireland) as previously described [39,41]. The sodium methoxide method was used to prepare fatty acid methyl esters (FAMEs) of the FAs present in the glycerolipids of the plasma TAG and PL fractions [42]. In short, lipids were suspended in dry ethyl ether, followed with the addition of methyl acetate and sodium methoxide, and then, the reaction was stopped by adding acetic acid after 5 min of incubation. Total lipids from the liver, brain, testis, eye, lung, and heart were analyzed with the acid-catalyzed method known to methylate all types of lipids [43] by overnight incubation of lipids with acetyl chloride and methanol at 50 °C. The fecal lipids were methylated with the sodium methoxide method and acid-catalyzed method to differentiate the glycerol-bound FAs from the free FAs.

### 2.4. Fatty Acid Composition

Gas chromatography was used to determine the fatty acid composition of the organs as described in [38]. Briefly, the fatty acid composition was determined with a Nexis GC-2030 (Shimadzu, Kyoto, Japan) with a flame ionization detector. The carrier gas was helium. The FAMEs were analyzed based on the area under the curve calculated using LabSolutions software. Fatty acid identification was based on the external standards (Supelco 37 Component FAME mix (Supelco, St. Louis, MO, USA), GLC-68D (Nu-Check-Prep, Elysian, MN, USA), GLC-490 (Nu-Check-Prep, Elysian, MN, USA), and 11A (Nu-Check-Prep, Elysian, MN, USA)). The quantification of the liver TAG, plasma TAG, brain, eye, testis, visceral fat, heart, lung, and feces was based on the triheptadecanoin, and that of liver PL and plasma PL was based on the dinonadecanoylphosphatidylcholine. The compositional data were calculated using the correction factors from external standards and expressed as relative % of all FAs for plasma, feces, liver, brain, eye, testis, visceral fat, lung, and heart and as µg/100 mg of individual FA for feces. The compositions of the feces and plasma of the (n-3)-adequate group have been described also previously as the normal feed group [38].

### 2.5. Statistical Analysis

Differences between the two feeding groups were evaluated with independent samples *t*-test performed using SPSS 25 program (IBM, Armonk, NY, USA). All data (except eye, which was analyzed once) were reported as means of duplicates ± standard deviation (SD), and the statistical significance was determined at *p* < 0.05.

## 3. Results

### 3.1. Body Weight and Organ Weight

There was no significant difference between the two experimental groups in the body weights of the rats at the beginning or end of the experiments (Table 3). The average weight gain in the rats of two groups was 269.88 ± 6.01 g. There were no significant differences in the organ weights between the two experimental groups (Table 3).

### 3.2. Tissue Total Lipids

There were no significant differences found between the two experimental groups in the total lipid content of any of the tissues analyzed (Table 4). The total lipid content in the visceral fat was highest among the rat organs studied in both experimental groups. In both experimental groups, there were 1.5–2 times more PLs than TAGs in the liver.

### 3.3. Fecal Lipids

The total fecal lipid and FA levels (µg/100 mg) of rat feces collected during and pooled from the last five days of the experiment are shown in Table 5. Total fecal lipids were 8–10-fold higher than glycerol-bound FAs, and this was seen equally in all FAs. Loss of fecal lipids, as total FAs, was higher in the (n-3)-deficient group than in the (n-3)-adequate group. The levels of ARA and docosatetraenoic acid (22:4(n-6), DTA) were significantly higher in the feces of the (n-3)-deficient group as compared to the (n-3)-adequate group, whereas EPA and DHA content was significantly lower in the feces of the (n-3)-deficient group than (n-3)-adequate group. Content of (n-3) FA as glycerol-bound FAs was equal in both experimental groups, while the total content of (n-3) PUFAs was significantly higher in the feces of the (n-3)-adequate group compared to the (n-3)-deficient group. However, the total (n-6) FA content was almost equal in glycerol-bound fatty acids and total fatty acids of both experimental groups. The level of glycerol-bound LA was significantly higher in the feces of the (n-3)-adequate group than that in the (n-3)-deficient group, whereas there was no significant difference found in the total FAs. The ALA content in the (n-3)-adequate group was significantly higher than the (n-3)-deficient group of glycerol-bound fatty acids content and total FA content.

### 3.4. Plasma Lipids

The FA composition of TAG and PL of fasting plasma reflected the FA profile of feed, and this was seen especially in the contents of oleic acid (18:1(n-9), OA), LA, and ALA (Table 6). Fasting plasma contained two to three times more PLs than TAGs. There were no differences in the contents of TAGs or PLs between the two groups (Table 6). The levels of EPA and DHA were significantly lower in the (n-3)-deficient group in fasting plasma TAGs and PLs as compared to the (n-3)-adequate group. On the contrary, the LC (n-6) PUFAs, such as ARA in the plasma, PLs, and DTA in both TAGs and PLs, were found to be significantly higher in the (n-3)-deficient group as compared to the (n-3)-adequate group. Taken together, the total (n-3) PUFA content in plasma TAGs and PLs of the (n-3)-adequate group was significantly higher than that of the (n-3)-deficient group.

### 3.5. Tissue EPA, Docosapentaenoic Acid (22:5(n-3), DPA), DHA, and Total (n-3) Fatty Acids

**Figure 1:**
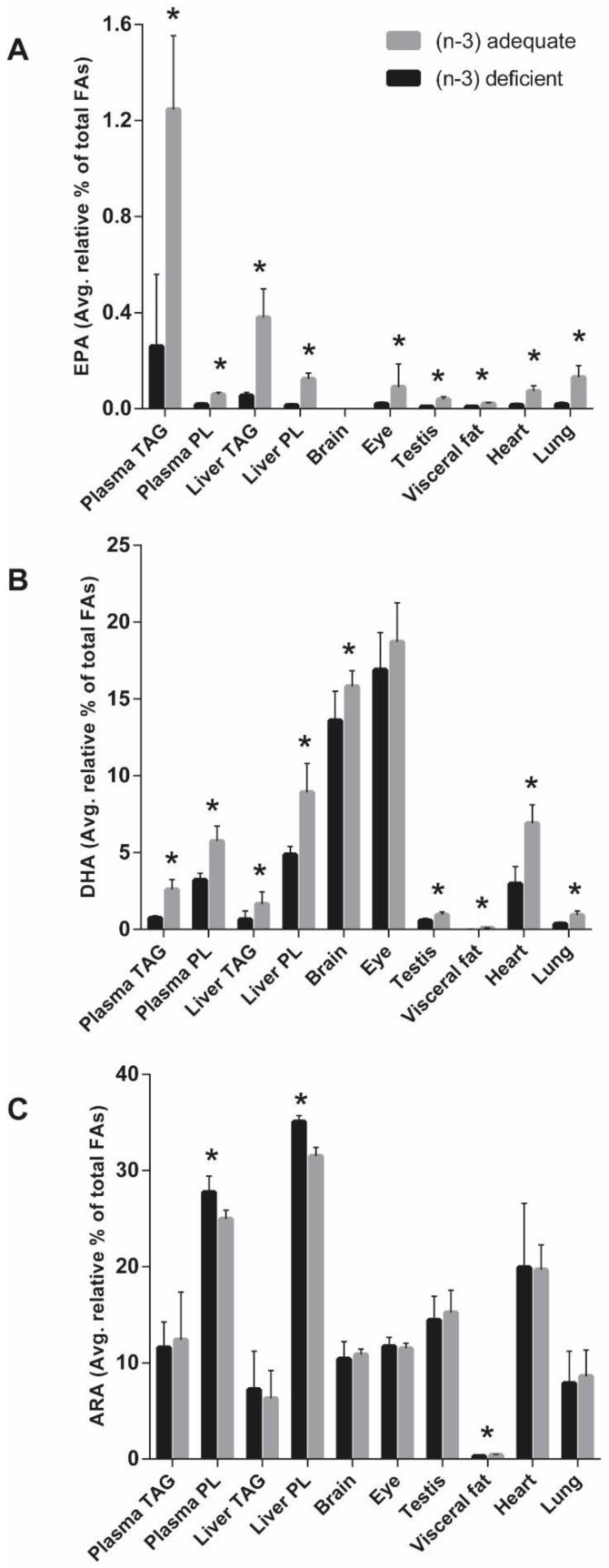
Average relative % (+/−SD) of (**A**) EPA, (**B**) DHA and (**C**) ARA of total fatty acids in (n-3)-deficient (*n* = 11) and (n-3)-adequate group (*n* = 12) of rat organs and plasma after 33 days of the feeding of (n-3)-deficient or (n-3)-adequate diet. The statistical difference (*p* < 0.05) between the diet groups is indicated by the asterisk.

The liver TAG and PL as well as the eye, testis, visceral fat, heart, and lung of the (n-3) adequate group had a significantly higher level of EPA as compared to the (n-3)-deficient group except for the brain, where the EPA was very low and almost undetectable (Table 7, Table 8 and Table 9). The liver TAG had the highest level of EPA (0.38% of total fatty acids), whereas the visceral fat had the lowest EPA (0.03% of total fatty acids) content in the (n-3)-adequate group (Figure 1). The range of EPA content among the tissues in the (n-3)-deficient group was 0.01–0.06% of total fatty acids, being the highest in the liver TAG. The DPA in the liver TAG, heart, and lung of the (n-3)-adequate group was significantly higher than in the corresponding tissues in the (n-3)-deficient group, whereas, in the liver PLs, it was non-significantly higher in the (n-3)-adequate group than (n-3)-deficient group. The highest level of DPA was found in the rat heart tissue of both experimental groups, being 0.89% and 0.27% of total fatty acids in the (n-3)-adequate group and (n-3)-deficient group, respectively. DPA was not detected in the rat brain, eye, testis, and visceral fat. All the organ tissues showed significantly higher DHA in the (n-3)-adequate group than (n-3)-deficient group, except the eye (Figure 1). The DHA content in the organs was in the range of 0.02–16.9% of total fatty acids in the (n-3)-deficient group and 0.01–18.7% in the (n-3)-adequate group. The rat eye was the richest in DHA in both experimental groups among the studied organs, with more DHA in the (n-3)-adequate group (18.7% of total fatty acids) than in the (n-3)-deficient group (16.9% of total fatty acids). However, this difference was not statistically significant (*p* = 0.95) between the two experimental groups. The visceral fat had the lowest DHA levels in both experimental groups among all rat organs. The brain tissue was the second highest after the eye in the richness of DHA, with a significantly higher level of DHA in the (n-3)-adequate group (15.8% of total fatty acids) compared to the (n-3)-deficient group (13.9% of total fatty acids). The DHA level of visceral fat of (n-3)-deficient group was 14% of the visceral fat of (n-3)-adequate group, being most affected by the (n-3)-deficient diet feeding, whereas the brain DHA was least affected with 86% of DHA level of the (n-3)-deficient group (Table 10). The rat liver TAG and PL, brain, testis, visceral fat, heart, and lung showed significantly higher total (n-3) FA content in the (n-3)-adequate group, whereas the eye had a non-significantly higher total (n-3) content in the (n-3)-adequate group compared to the (n-3)-deficient group. The eye had the highest and the visceral fat had the lowest total (n-3) FA in the (n-3)-deficient group among all rat organs.

### 3.6. Tissue ARA, DTA, and Total (n-6) Fatty Acids

The two experimental diets resulted in almost equal amounts of ARA in the liver TAG, brain, eye, testis, heart, and lung. The liver PLs of the (n-3)-deficient group had significantly higher ARA levels than that of the (n-3)-adequate group. Visceral fat of the (n-3)-adequate group had significantly higher ARA levels than that of the (n-3)-deficient group (Table 7, Table 8 and Table 9). The liver PLs had the highest ARA (31–35% of total fatty acids), whereas the visceral fat had the lowest ARA (0.3–0.4% of total fatty acids) in both the experimental groups. The (n-3)-deficient group of liver TAGs and PLs, brain, eye, heart, and lung had significantly more DTA as compared to the (n-3)-adequate group (Figure 1). The level of DTA in the rat testis and visceral fat did not differ between both experimental groups. However, the DTA content of rat testis was the highest (11% of total fatty acids), and the visceral fat had the lowest DTA content (0.04% of total fatty acids) among all analyzed organs in both experimental groups. The total (n-6) FA content varied among rat organs, showing significantly higher levels in the (n-3)-adequate group of liver TAG, testis, visceral fat, and lung; significantly higher levels in liver PL of the (n-3) deficient group; and almost equal levels in the brain, eye, and heart of both experimental groups.

## 4. Discussion

In this study, rats were fed an (n-3)-deficient diet for 33 days after one week of adaptive feeding post-weaning. The study aimed to investigate the impact of (n-3)-deficient diet in the form of peanut-oil-based rodent chow on the levels of LC (n-3) PUFAs in various tissues and organs in order to understand the extent of LC (n-3) PUFAs depletion as compared to (n-3)-adequate diet in the form of soybean-oil-based rodent chow in young adult rats. In terms of animal models, the rodent model was chosen because of the practical considerations of its handling, housing, maintenance, and relatively short generation time over primates or pigs. Results of this study can be used also to evaluate the conditions needed to reach mild (n-3) deficiency in the first generation of (n-3)-deficient rats and to evaluate the feasibility to collect data from a variety of organs or only selected ones.

The fecal FA composition reflected that of the feed. The major contributors of fecal FAs in both experimental groups were saturated FAs, particularly 16:0 and 18:0, excreted in the form of calcium and magnesium soaps as described previously [44,45]. The higher EPA loss in feces as total FAs in the (n-3)-adequate group as compared to (n-3)-deficient group might suggest less EPA in the intestinal epithelial cells. Differences in microbial metabolism of the available FAs or the anatomical and physiological features of the gastro-intestinal tract might have affected this outcome [46]. However, the similar loss of DHA in both experimental groups could be an indication that that DHA excreted to the digestive tract is more efficiently reabsorbed than EPA or ARA. The higher fecal ARA in the total FAs of the (n-3)-deficient group could be an indication that the (n-3) FAs were compensated by ARA in the epithelial cells. To the best of our knowledge, this is the first time when the fecal samples are included in a (n-3)-deficiency study.

The plasma FA levels in the present study were indicative of the tissue LC (n-3) PUFAs across a range of rat organ tissues. Similarly, a correlation between plasma FAs and those in liver, heart, and quadriceps muscle have been previously reported in rats after the dietary treatment with ALA for three weeks [47]. The decrease in LC (n-3) PUFAs (mainly EPA and DHA) in the organs of the (n-3)-deficient group was found to have been compensated with an increase in the LC (n-6) PUFAs (mainly ARA) predominantly in liver PL followed by the heart among all analyzed organs. The balance between (n-3) and (n-6) FAs is important as the interaction between them affects their further metabolism and incorporation into the tissues and production of, e.g., eicosanoids and docosanoids [27].

The incorporation of ALA to rat organs, except for the brain and eye, was significantly higher in the (n-3)-adequate group when compared to the (n-3)-deficient group. The ALA content of the brain and eye in both experimental groups was almost equal, which indicates that ALA level of the eyes or brains are kept at a steady level despite acute (n-3) deficiency, at least in cases where normal levels of (n-3) were available during pregnancy and weaning. A significant proportion of dietary ALA is taken up by the visceral fat [48], and this was seen in the visceral fat of the (n-3)-adequate group also in the present study. In the presence of ALA in the (n-3)-adequate group, the lower levels of longer-chain metabolites (EPA and DHA) suggest that the visceral fat efficiently takes up ALA but does not efficiently convert ALA to EPA and DHA [49]. However, the comparatively low ALA in the (n-3)-adequate group of other tissues is likely to be the result of β- oxidation of ALA to CO_2_ [50]. Analysis of the brain revealed that the DHA content of the (n-3)-deficient group was 86% of the DHA content of the (n-3)-adequate group. This suggests that 33 days of (n-3)-deficient feeding was enough to induce DHA deficiency. However, the eye DHA did not differ significantly between the groups, indicating that the eye DHA in the (n-3)-deficient rats was maintained by the (n-3)-adequate diet during pregnancy and weaning. A previous study showed that single-generation (n-3) deprivation in rats for 18 weeks reduced 36% of DHA in the brain [15], whereas (n-3) deprivation in the present study lasted for 33 days and resulted in milder (n-3) deficiency in the brain. Another study in rats showed lower DHA in brain cells and organelles after feeding sunflower oil, which is low in ALA as compared to soybean oil [32]. However, the dams in their study were fed either the sunflower- or soybean-oil-based diet for two generations, and the (n-3) deficiency was studied in the following generation of rats. Retinal DHA of 23% was reported in guinea pigs after safflower-oil-based feeding as compared to canola oil for 24 weeks [21]. This study indicated that the loss of DHA in the first 12 weeks was greater than that of the second 12 weeks, implying that the retinal DHA was altered by the ALA supply in the early weeks of life. However, the observed eye DHA of both experimental groups in the present study was not affected in 33 days, and a longer intervention is thus needed to achieve (n-3) deprivation in the eye of rats.

We observed lower DHA levels in the liver, brain, testis, visceral fat, heart, and lung and the lower EPA levels in the liver, eye, testis, visceral fat, heart, and lung of the (n-3)-deficient group as compared to (n-3)-adequate group. This can be explained by the lack of ALA in the diet of (n-3)-deficient group for the EPA and DHA synthesis *in vivo* [35,51,52]. There are very few studies involving heart, lung, and testis to determine the effect of (n-3) deficiency. A previous study showed a decline in the DHA level of heart and testes in the first three weeks of intervention and remained nearly stable thereafter with (n-3)-deficient feeding in 60-day-old rats from the third generation [31]. Low levels of DHA were reported in the heart, lung, and testis of rats from the third generation fed with sunflower oil [32] and lung and testis of rats from the fourth generation [33] together with other organs as compared to soybean or rapeseed oil feeding. Our results concerning (n-3) FAs are in agreement with previous reports; however, the animals studied in the past studies were from third or fourth generations, unlike our present study. The liver has a higher activity of desaturation and elongation than the brain [17], suggesting that most of the observed DHA of the brain and eye in the present study was derived from the liver. The feeding of (n-3)-adequate diet resulted in markedly higher DHA and DPA distribution in the heart and lung as compared to the testis, which could be a result of a greater magnitude of uptake from the plasma lipid pool. The ALA cannot be converted to DHA in the rat heart because of the lack of elongase-2 [53]. However, the FAs as TAGs and PLs from circulating lipoproteins can enter the heart to a measurable extent through very low-density lipoprotein receptors [54,55]. The DHA in the heart and lung was much higher than the visceral fat DHA, which suggests that DHA is not prioritized for energy storage purposes in the visceral fat perhaps because DHA oxidizes easily.

Although the (n-3)-adequate diet increased LC (n-3) PUFAs as compared to (n-3)-deficient diet, as expected in the present study, it is known that the metabolism of (n-3) FAs depends also on the (n-6) FAs due to the competition of (n-3) and (n-6) FAs for the same enzymes [56]. ARA is as important as DHA in normal brain growth [57]. The present study showed that the ALA-deficient diet increased ARA in the liver PL and supports the idea that ALA inhibits the conversion of LA into LC (n-6) PUFAs [58]. Previously perinatal dietary (n-3) fatty acid depletion and subsequent repletion with the (n-3) fatty acid (ALA) in rats showed that chronic (n-3) FA-deficiency upregulated liver (n-6) FA biosynthesis and increased membrane ARA composition [29]. These findings were confirmed in the follow-up study [28]. Other peripheral organs in this study had an almost equal level of ARA and were not affected by the ALA deficiency, suggesting that ARA may be relatively resistant to large changes in the ratio of (n-3) to (n-6) FAs in the diet. The observed higher ARA in the heart as compared to brain, eye, testis, visceral fat, and lung suggests greater uptake of ARA by the heart after being delivered by the liver. The LA level from the feed was reflected in the organs in both experimental groups. The lower levels of LC (n-3) PUFAs in the (n-3)-deficient group were offset by the increase in the LC (n-6) PUFAs, especially DTA, in addition to ARA, in the liver, brain, eye, heart, and lung. The observed significant increase in the LC (n-6) PUFAs of the (n-3)-deficient group was also previously seen in the form of 22:5(n-6) of rat brain phospholipid [15,59], 22:5(n-6) of rat heart [53], DTA of liver of growing pigs [58], and 22:5(n-6) in retinal and brain of rhesus monkeys [24]. In this study, we did not detect and quantify 22:5(n-6) but also studying the relationship of DHA and DPA with 22:5(n-6) might be of interest in the following studies. However, the total PUFA content in the brain and eye of the (n-3)-deficient and (n-3)-adequate groups remained approximately equal after 33 days of the experiment as observed earlier in brain PLs in an even longer intervention of 16 weeks with safflower and soybean oil in rats born to dams kept on the same diet six weeks before mating and during gestation [59]. The largest extent of DTA accumulation in testis among all organs might suggest local elongation or desaturation of the (n-6) FAs.

While these data are derived from the first-generational (n-3)-deficient rat model, the reductions in the levels of LC (n-3) PUFAs are not as large as multigenerational studies with longer experimental duration [37]. However, brain DHA content in the (n-3)-deficient group was about 86% of the brain DHA content of the (n-3)-adequate groups, and it is a significant sign of (n-3) mild deficiency, which was induced in a period of only 33 days. Our study showed that the visceral fat was highly affected by the (n-3) deficiency, followed by liver TAG, lung, heart, liver PL, testis, eye, and brain. The effect of (n-3)-deficient and (n-3)-adequate feeding on the accumulation of LC (n-3) PUFAs and LC (n-6) PUFAs was evident in the FA lipid composition of various rat organs. The (n-3) deficiency mainly occurs because of the low intake or absence of seafood in the diet, use of cooking oils lacking ALA, or poor maternal and infant nutrition. The present study had an adequate amount of LA in both experimental groups and included peanut oil as an (n-3)-deficient source of FA and soybean oil as an (n-3)-adequate source of FAs. This kind of situation is common in the real life. Thus, the first-generation (n-3)-deficient rat model is clinically relevant to study the (n-3) deficiency in young animals in conditions that could mimic the human LC (n-3) PUFA status.

## Figures and Tables

**Table 1:** Composition of diets.

Ingredients	Contents (g/kg)
Corn starch	397
Casein (>85% protein)	200
Corn dextrin (90–94% tetrose)	132
Sucrose	100
Oil ^1^	70
Fiber	50
Minerals	35.5
Vitamins	10
l-cystinol	3
Choline bitartrate (42% choline)	2.5
*Tert*-butylhydroquinone	0.014

^1^ Modified AIN-93G low-(n-3) FA diet for the (n-3)-deficient group contained peanut oil, and standard (n-3) AIN-93G diet for (n-3)-adequate group contained soybean oil.

**Table 2:** Fatty acid composition of oils used in the feed.

Fatty Acid	Peanut Oil ^1^	Soybean Oil ^2^
16:0	10.3	10.8
18:0	3.2	4.4
18:1(n-9), OA	45.6	22.7
18:2(n-6), LA	32.3	51.9
18:3(n-3), ALA	0.2	6.3
20:0	1.4	0.4
22:0	2.9	0.4
24:0	1.4	0.1
Others ^3^	2.8	3.0

Values are expressed as mean mass percentages of two replicates. ^1^ Peanut oil was used in the feed of the (n-3)-deficient group. ^2^ Soybean oil was used in the feed of the (n-3)-adequate group. ^3^ This category includes 16:1(n-7), 18:1(n-7), 18:3(n-6), 20:1(n-9), and 23:0. OA, oleic acid; LA, linoleic acid; ALA, α-linolenic.

**Table 3:** The organ weights and body weight (g) of the rats at the end of the experiment.

Groups ^1^	(n-3) Deficient	(n-3) Adequate
Eye	0.3 ± 0.02	0.3 ± 0.02
Brain	2.1 ± 0.1	2.0 ± 0.2
Liver	11.7 ± 1.4	12.5 ± 1.5
Heart	1.4 ± 0.2	1.3 ± 0.1
Lung	1.6 ± 0.2	1.5 ± 0.2
Testis	2.9 ± 0.3	3.0 ± 0.3
Visceral Fat	5.1 ± 1.7	5.2 ± 1.6
Total body weight	366 ± 26	364 ± 35

^1^ The (n-3)-deficient and (n-3)-adequate groups received peanut-oil-based (n-3) FA-deficient diet and soybean-oil-based AIN-93G standard diet, respectively, for 33 days. Values for organ weight and total body weight are mean (g) ± SD; *n* = 12 for (n-3)-adequate group and *n* = 11 for (n-3)-deficient group.

**Table 4:** Total lipid (µg/100 mg) of rat organ tissues at the end of the experiment.

	Groups ^1^	
Organs	(n-3) Deficient	(n-3) Adequate
Liver TAG	251.7 ± 92.9	200.2 ± 42.5
Liver PL	419.0 ± 37.7	439.7 ± 19.6
Brain	467.1 ± 24.9	479.5 ± 17.7
Eye	603.6 ± 77.4	551.2 ± 45.0
Testis	195.5 ± 39.0	181.7 ± 33.5
Heart	301.5 ± 153.1	233.1 ± 24.6
Lung	422.2 ± 158.7	360.1 ± 103.7
Visceral fat	2957.2 ± 710.6	2773.7 ± 625.9

^1^ The (n-3)-deficient and (n-3)-adequate groups received peanut-oil-based (n-3) FA-deficient diet and soybean-oil-based AIN-93G standard diet, respectively, for 33 days. Values for total lipid are mean ± SD, *n* = 12 for (n-3)-adequate group and *n* = 11 for (n-3)-deficient group. TAG, triacylglycerol; PL, phospholipid.

**Table 5:** Glycerol-bound fatty acids and total (free and esterified) fatty acids (µg/100 mg) excreted in the rat feces collected during the last 5 days of the experiment.

Fatty Acids	Groups ^1^			
	(n-3) Deficient	(n-3) Adequate	(n-3) Deficient	(n-3) Adequate
	Glycerol-Bound Fatty Acids		Total Fatty Acids	
18:1(n-9), OA	18.84 ± 4.51 ^a^	12.66 ± 1.76 ^b^	186.96 ± 99.24 ^a^	83.63 ± 17.57 ^b^
18:2(n-6), LA	6.31 ± 0.92 ^a^	7.56 ± 1.25 ^b^	58.28 ± 28.81	63.99 ± 16.89
18:3(n-3), ALA	0.2 ± 0.1 ^a^	0.42 ± 0.06 ^b^	1.14 ± 0.84 ^a^	4.64 ± 1.31 ^b^
20:4(n-6), ARA	1.96 ± 0.29	1.72 ± 0.53	7.13 ± 1.2 ^a^	5.18 ± 1.24 ^b^
20:5(n-3), EPA	0.16 ± 0.08	0.09 ± 0.05	0.09 ± 0.05 ^a^	0.16 ± 0.07 ^b^
22:4(n-6), DTA	0.71 ± 0.3	0.93 ± 0.46	1.31 ± 0.84 ^a^	0.44 ± 0.15 ^b^
22:5(n-3), DPA	0.02 ± 0.02	0.01 ± 0	0.21 ± 0.1	0.17 ± 0.09
22:6(n-3), DHA	0.13 ± 0.05	0.17 ± 0.04	0.62 ± 0.21	0.77 ± 0.26
Total (n-6)	10.25 ± 1.27	11.44 ± 1.93	79.64 ± 30.66	80.62 ± 18.93
Total (n-3)	1.19 ± 0.2	1.38 ± 0.28	4.96 ± 1.45 ^a^	7.62 ± 1.56 ^b^
Total PUFA	11.45 ± 1.24	12.83 ± 2.17	84.6 ± 31.79	88.24 ± 20.42
TL (µg/100 mg)	141.6 ± 16.9	127.5 ± 21.5	1635.0 ± 364.0 ^a^	1098.0 ± 196.9 ^b^

^1^ The (n-3)-deficient and (n-3)-adequate groups received peanut-oil-based (n-3) FA-deficient diet and soybean-oil-based AIN-93G standard diet, respectively, for 33 days. Values are mean (µg/100 mg) ± SD, *n* = 12 for (n-3)-adequate group and *n* = 11 for (n-3)-deficient group. Values with different superscripts indicate significant differences between the (n-3)-deficient and (n-3)-adequate feedings (*p* < 0.05). PUFA, polyunsaturated fatty acids; TL, total lipids; OA, oleic acid; LA, linoleic acid; ALA, α-linolenic; ARA, arachidonic acid; EPA, eicosapentaenoic acid; DTA, docosatetraenoic acid; DPA, docosapentaenoic acid; DHA, docosahexaenoic acid. Detailed FA composition is presented in Supplementary Table S1.

**Table 6:** Fatty acids of the TAG and PL fractions of fasting rat plasma (% of total fatty acids) of (n-3)-deficient and (n-3)-adequate groups.

Fatty Acids	Groups ^1^			
	(n-3) Deficient	(n-3) Adequate	(n-3) Deficient	(n-3) Adequate
	TAG		PL	
18:1(n-9), OA	28.32 ± 3.3 ^a^	19.81 ± 2.3 ^b^	5.18 ± 0.5 ^a^	3.66 ± 0.3 ^b^
18:2(n-6), LA	22.68 ± 1.98 ^a^	28.24 ± 2.87 ^b^	10.72 ± 1.56 ^a^	12.97 ± 1.52 ^b^
18:3(n-3), ALA	0.5 ± 0.17 ^a^	2.19 ± 0.35 ^b^	0.02 ± 0.01 ^a^	0.04 ± 0 ^b^
20:4(n-6), ARA	11.59 ± 2.64	12.39 ± 4.96	27.72 ± 1.69 ^a^	24.93 ± 0.91 ^b^
20:5(n-3), EPA	0.25 ± 0.29 ^a^	1.24 ± 0.3 ^b^	0.01 ± 0 ^a^	0.05 ± 0 ^b^
22:4(n-6), DTA	0.99 ± 0.32 ^a^	0.33 ± 0.19 ^b^	1.66 ± 0.4 ^a^	0.32 ± 0.15 ^b^
22:5(n-3), DPA	0.12 ± 0.05	0.09 ± 0.1	0.02 ± 0.01	0.01 ± 0
22:6(n-3), DHA	0.73 ± 0.15 ^a^	2.58 ± 0.66 ^b^	3.2 ± 0.44 ^a^	5.74 ± 0.97 ^b^
Total (n-6) FA	36.59 ± 2.17 ^a^	42.1 ± 3.67 ^b^	41.14 ± 0.66 ^a^	39.67 ± 1.1 ^b^
Total (n-3) FA	1.61 ± 0.36 ^a^	6.1 ± 0.56 ^b^	3.27 ± 0.43 ^a^	5.87 ± 0.96 ^b^
Total PUFA	38.21 ± 2.35 ^a^	48.21 ± 3.74 ^b^	44.41 ± 0.75 ^a^	45.54 ± 0.68 ^b^
TL (µg/100 mg)	54.8 ± 25.1	40.5 ± 16.5	127.7 ± 16.5	125.7 ± 21.0

^1^ The (n-3)-deficient and (n-3)-adequate groups received peanut-oil-based (n-3) FA-deficient diet and soybean-oil-based AIN-93G standard diet, respectively, for 33 days. Values are mean (relative % of total FAs) ± SD, *n* = 12 for (n-3)-adequate group and *n* = 11 for (n-3)-deficient group. Values with different superscripts indicate significant differences between the (n-3)-deficient and (n-3)-adequate feedings (*p* < 0.05). PUFA, polyunsaturated fatty acids; TL, total lipids; OA, oleic acid; LA, linoleic acid; ALA, α-linolenic; ARA, arachidonic acid; EPA, eicosapentaenoic acid; DTA, docosatetraenoic acid; DPA, docosapentaenoic acid; DHA, docosahexaenoic acid. Detailed FA composition is presented in Supplementary Table S2.

**Table 7:** Fatty acids of the TAG and PL fractions of rat liver (% of total fatty acids) of (n-3)-deficient and (n-3)-adequate groups.

Fatty Acids	Groups ^1^			
	(n-3) Deficient	(n-3) Adequate	(n-3) Deficient	(n-3) Adequate
	TAG		PL	
18:1(n-9), OA	34.24 ± 4.42 ^a^	24.18 ± 2.33 ^b^	4.5 ± 0.41 ^a^	3.42 ± 0.36 ^b^
18:2(n-6), LA	19.76 ± 2.41 ^a^	26.96 ± 6.28 ^b^	9.68 ± 1.11 ^a^	11.65 ± 1.79 ^b^
18:3(n-3), ALA	0.27 ± 0.06 ^a^	1.7 ± 0.49 ^b^	0.03 ± 0.02 ^a^	0.11 ± 0.04 ^b^
20:4(n-6), ARA	7.22 ± 4.01	6.29 ± 2.94	35.05 ± 0.68 ^a^	31.51 ± 0.91 ^b^
20:5(n-3), EPA	0.06 ± 0.02 ^a^	0.38 ± 0.13 ^b^	0.02 ± 0.01 ^a^	0.13 ± 0.03 ^b^
22:4(n-6), DTA	0.65 ± 0.37 ^a^	0.2 ± 0.09 ^b^	2.4 ± 0.61 ^a^	0.45 ± 0.23 ^b^
22:5 (n-3), DPA	0.02 ± 0.02 ^a^	0.03 ± 0.02 ^b^	0.08 ± 0.03	0.1 ± 0.05
22:6(n-3), DHA	0.66 ± 0.56 ^a^	1.66 ± 0.8 ^b^	4.86 ± 0.55 ^a^	8.92 ± 1.88 ^b^
Total (n-6)	28.21 ± 3.86 ^a^	34.22 ± 5.64 ^b^	48.15 ± 0.63 ^a^	44.95 ± 2.08 ^b^
Total (n-3)	1.47 ± 0.69 ^a^	4.11 ± 0.74 ^b^	5.71 ± 0.58 ^a^	9.62 ± 1.86 ^b^
Total PUFA	29.68 ± 4.42 ^a^	38.32 ± 6.04 ^b^	53.85 ± 0.66 ^a^	54.56 ± 0.72 ^b^

^1^ The (n-3)-deficient and (n-3)-adequate groups received peanut-oil-based (n-3) FA-deficient diet and soybean-oil-based AIN-93G standard diet, respectively, for 33 days. Values are mean (relative % of total FAs) ± SD, *n* = 12 for (n-3)-adequate group and *n* = 11 for (n-3)-deficient group. Values with different superscripts indicate significant differences between the (n-3)-deficient and (n-3)-adequate feedings (*p* < 0.05). PUFA, polyunsaturated fatty acids; OA, oleic acid; LA, linoleic acid; ALA, α-linolenic; ARA, arachidonic acid; EPA, eicosapentaenoic acid; DTA, docosatetraenoic acid; DPA, docosapentaenoic acid; DHA, docosahexaenoic acid. Detailed FA composition is presented in Supplementary Table S3.

**Table 8:** Fatty acid composition of the rat brain, eye, and testis (% of total fatty acids) of (n-3)-deficient and (n-3)-adequate groups.

Fatty Acids	Groups ^1^					
	Brain		Eye		Testis	
	(n-3) Deficient	(n-3) Adequate	(n-3) Deficient	(n-3) Adequate	(n-3) Deficient	(n-3) Adequate
18:1(n-9), OA	14.85 ± 0.85	14.49 ± 0.75	15.85 ± 2.69 ^a^	13.21 ± 1.62 ^b^	17.25 ± 3.89 ^a^	12.48 ± 2.08 ^b^
18:2(n-6), LA	0.61 ± 0.08 ^a^	0.74 ± 0.09 ^b^	5.28 ± 1.94	5.75 ± 1.86	7.77 ± 1.97	9.96 ± 3.08
18:3(n-3), ALA	0.02 ± 0.01	0.02 ± 0.01	0.13 ± 0.24	0.24 ± 0.12	0.14 ± 0.07 ^a^	0.62 ± 0.32 ^b^
20:4(n-6), ARA	10.39 ± 1.84	10.86 ± 0.6	11.72 ± 0.95	11.5 ± 0.57	14.45 ± 2.49	15.24 ± 2.35
20:5(n-3), EPA	nd ^2^	nd ^2^	0.02 ± 0.01 ^a^	0.1 ± 0.1 ^b^	0.01 ± 0.01 ^a^	0.04 ± 0.02 ^b^
22:4(n-6), DTA	1.45 ± 0.3 ^a^	0.73 ± 0.34 ^b^	1.51 ± 0.34 ^a^	0.54 ± 0.1 ^b^	11.26 ± 1.89	11.55 ± 1.76
22:5(n-3), DPA	nd ^2^	nd ^2^	nd ^2^	nd ^2^	nd ^2^	nd ^2^
22:6(n-3), DHA	13.59 ± 1.93 ^a^	15.8 ± 1.04 ^b^	16.9 ± 2.42	18.71 ± 2.55	0.58 ± 0.14 ^a^	0.94 ± 0.21 ^b^
Total (n-6)	13.11 ± 1.74	13.01 ± 0.7	19.28 ± 1.45	18.63 ± 1.88	34.8 ± 2.56 ^a^	38.2 ± 1.08 ^b^
Total (n-3)	16.55 ± 2.39 ^a^	18.64 ± 1.01 ^b^	18.93 ± 2.48	20.7 ± 2.46	2.46 ± 0.35 ^a^	3.39 ± 0.16 ^b^
Total PUFA	29.66 ± 4.08	31.65 ± 1.33	38.21 ± 2.04	39.32 ± 1.39	37.26 ± 2.87 ^a^	41.59 ± 1.14 ^b^

^1^ The (n-3)-deficient and (n-3)-adequate groups received peanut-oil-based (n-3) FA-deficient diet and soybean-oil-based AIN-93G standard diet, respectively, for 33 days. Values are mean (relative % of total FAs) ± SD, *n* = 12 for (n-3)-adequate group and *n* = 11 for (n-3)-deficient group. Values with different superscripts indicate significant differences between the (n-3)-deficient and (n-3)-adequate feedings (*p* < 0.05). PUFA, polyunsaturated fatty acids; ^2^ nd, not detected; OA, oleic acid; LA, linoleic acid; ALA, α-linolenic; ARA, arachidonic acid; EPA, eicosa-pentaenoic acid; DTA, docosatetraenoic acid; DPA, docosapentaenoic acid; DHA, docosahexaenoic acid. Detailed FA composition is presented in Supplementary Table S4.

**Table 9:** Fatty acid composition of the rat visceral fat, heart, and lung (% of total fatty acids) of (n-3)-deficient and (n-3)-adequate groups.

Fatty Acids	Groups ^1^					
	Visceral Fat		Heart		Lung	
	(n-3) Deficient	(n-3) Adequate	(n-3) Deficient	(n-3) Adequate	(n-3) Deficient	(n-3) Adequate
18:1(n-9), OA	10.35 ± 4.1	11.01 ± 3.37	14.78 ± 8.71 ^a^	7.79 ± 2.61 ^b^	28.49 ± 6.34 ^a^	20.14 ± 4.09 ^b^
18:2(n-6), LA	20.85 ± 1.04 ^a^	30.92 ± 2.53 ^b^	18.12 ± 2 ^a^	23.29 ± 1.84 ^b^	12 ± 2.41 ^a^	15.8 ± 3.63 ^b^
18:3(n-3), ALA	0.37 ± 0.06 ^a^	2.59 ± 0.21 ^b^	0.08 ± 0.05 ^a^	0.41 ± 0.18 ^b^	0.16 ± 0.05 ^a^	0.89 ± 0.26 ^b^
20:4(n-6), ARA	0.29 ± 0.05 ^a^	0.41 ± 0.08 ^b^	19.94 ± 6.65	19.68 ± 2.56	7.88 ± 3.35	8.62 ± 2.7
20:5(n-3), EPA	0.01 ± 0.01 ^a^	0.03 ± 0.01 ^b^	0.02 ± 0.01 ^a^	0.08 ± 0.03 ^b^	0.02 ± 0.01 ^a^	0.14 ± 0.05 ^b^
22:4(n-6), DTA	0.04 ± 0.02	0.04 ± 0.02	2.44 ± 1.19 ^a^	0.8 ± 0.22 ^b^	0.48 ± 0.23 ^a^	0.2 ± 0.08 ^b^
22:5(n-3), DPA	nd ^2^	nd ^2^	0.27 ± 0.11 ^a^	0.89 ± 0.23 ^b^	0.07 ± 0.04 ^a^	0.37 ± 0.15 ^b^
22:6(n-3), DHA	0.02 ± 0.01 ^a^	0.1 ± 0.03 ^b^	2.97 ± 1.12 ^a^	6.91 ± 1.22 ^b^	0.36 ± 0.13 ^a^	0.92 ± 0.29 ^b^
Total (n-6)	21.43 ± 1.03 ^a^	31.74 ± 2.48 ^b^	41.13 ± 9.09	44.55 ± 2.67	21.09 ± 1.99 ^a^	25.61 ± 2.53 ^b^
Total (n-3)	0.44 ± 0.06 ^a^	2.81 ± 0.22 ^b^	4.55 ± 1.44 ^a^	9.21 ± 1.36 ^b^	2.30 ± 0.87 ^a^	3.97 ± 0.8 ^b^
Total PUFA	21.87 ± 1.02 ^a^	34.55 ± 2.64 ^b^	45.7 ± 10.46 ^a^	53.75 ± 3.24 ^b^	23.39 ± 2.76 ^a^	29.57 ± 2.83 ^b^

^1^ The (n-3)-deficient and (n-3)-adequate groups received peanut-oil-based (n-3) FA-deficient diet and soybean-oil-based AIN-93G standard diet, respectively, for 33 days. Values are mean (relative % of total FAs) ± SD, *n* = 12 for (n-3)-adequate group and *n* = 11 for (n-3)-deficient group. Values with different superscripts indicate significant differences between the (n-3)-deficient and (n-3)-adequate feedings (*p* < 0.05). PUFA, polyunsaturated fatty acids; ^2^ nd, not detected; OA, oleic acid; LA, linoleic acid; ALA, α-linolenic; ARA, arachidonic acid; EPA, eicosapentaenoic acid; DTA, docosatetraenoic acid; DPA, docosapentaenoic acid; DHA, docosahexaenoic acid. Detailed FA composition is presented in Supplementary Table S5.

**Table 10:** Level of DHA in organs of (n-3)-deficient group, with values expressed as a percentage of the respective values in the (n-3)-adequate group.

Groups	Percentage ^1^
Visceral fat	14 ± 0.03
Liver TAG	37 ± 0.34
Lung	39 ± 0.14
Heart	43 ± 0.17
Liver PL	50 ± 0.07
Testis	56 ± 0.15
Eye	83 ± 0.13
Brain	87 ± 0.13

^1^ The values are expressed as a percentage ± SD; *n* = 12 for (n-3)-adequate group and *n* = 11 for (n-3)-deficient group. TAG, triacylglycerol; PL, phospholipid.

## Data Availability

The data presented in this study are available on reasonable request from the corresponding author.

## Supplementary Materials

The following supporting information can be downloaded at: https://www.mdpi.com/article/10.3390/foods11020208/s1, Figure S1: Animal trial model diagram showing the feeding of two groups and housing system during the experiment; Table S1: Glycerol-bound fatty acids and total (free and esterified) fatty acids (µg/100 mg) excreted in the rat feces collected during the last 5 days of the experiment; Table S2: Fatty acids of the TAG and PL fractions of fasting rat plasma (% of total fatty acids) of (n-3) deficient and (n-3) adequate groups; Table S3: Fatty acids of the TAG and PL fractions of rat liver (% of total fatty acids) of (n-3) deficient and (n-3) adequate groups; Table S4: Fatty acid composition of the rat brain, eye, and testis (% of total fatty acids) of (n-3) deficient and (n-3) adequate groups and Table S5: Fatty acid composition of the rat visceral fat, heart, and lung (% of total fatty acids) of (n-3) deficient and (n-3) adequate groups).
