# Correction: Kulkarni et al. Tissue-Specific Content of Polyunsaturated Fatty Acids in (n-3) Deficiency State of Rats. *Foods* 2021, *11*, 208

**DOI:** 10.3390/foods12173293

**Published:** 2023-09-01

**Authors:** Amruta Kulkarni, Ai Zhao, Baoru Yang, Yumei Zhang, Kaisa M. Linderborg

**Affiliations:** 1 Food Chemistry and Food Development, Department of Life Technologies, University of Turku, 20520 Turku, Finland; amruta.kulkarni@utu.fi (A.K.); bayang@utu.fi (B.Y.); 2 Vanke School of Public Health, Tsinghua University, Beijing 100083, China; xiaochaai@163.com; 3 Department of Nutrition & Food Hygiene, School of Public Health, Peking University Health Science Center, Beijing 100191, China

The authors would like to make the following corrections to a published paper [1]. The authors state that the scientific conclusions are unaffected. A list of corrections appears below. The authors would like to apologize for any inconvenience caused.

## 1. Text Correction

A correction: an addition of the methylation method of fatty acids for liver TAG, liver PL and visceral fat has been made to *2.3, Lipid Extraction, Fractionation into Neutral and Polar Rich Lipids, and Methylation of FAs*:

Fatty acids from the brain, testis, eye, lung, and heart were analyzed with the acid-catalyzed method known to methylate all types of lipids [43] via overnight incubation of lipids with acetyl chloride and methanol at 50 °C, whereas fatty acids from liver TAG, liver PL, and visceral fat were methylated with the sodium methoxide method. For clarity, (tissue and solvent) has been added in the extraction method of total lipids.

A correction has been made to *2.4, Fatty Acid Composition*. Additional external standards (GLC-566C, 22:3(n-3), 22:4(n-6), 22:5(n-6) have been listed:

Fatty acid identification was based on the external standards, GLC-68D, GLC-490, GLC-566C, 22:3(n-3), 22:4(n-6), 22:5(n-6) (Nu-Check-Prep, Elysian, MN, USA), and Supelco 37 Component FAME mix (Supelco, St. Louis, MO, USA).

A correction has been made to *3.2, Tissue Total Lipids*. A sentence has been added:

The total lipid content of the visceral fat is low and it is likely that not all fat was extracted.

A correction has been made to *3.3, Fecal Lipids:*

Docosatetraenoic acid (22:4(n-6), DTA) has been corrected to docosapentaenoic acid (22:5(n-6), DPAn-6).

A correction has been made to *3.4, Plasma Lipids*; Section *3.6 Tissue ARA, DTA, and Total (n-6) Fatty Acids*; and Section 4, Discussion:

DTA has been corrected to DPAn-6.

A correction has been made to *4, Discussion*. The following original sentence in the paragraph 6 has been removed:

“In this study, we did not detect and quantify 22:5(n-6) but also studying the relationship of DHA and DPA with 22:5(n-6) might be of interest in the following studies.”

## 2. Error in Table Legend

In the original publication, there was a mistake in the legends for Tables 5–9. Fatty acid DTA, docosatetraenoic acid, has been corrected to DPAn-6, docosapentaenoic acid.

## 3. Error in Table

In the original publication, there was a mistake in Tables 5–9. The correct identification of the fatty acid originally listed as 22:4(n-6) should be 22:5(n-6), DPAn-6.

In the original publication, there was a mistake in Table 4. There was a calculation error associated with a dilution factor. The corrected version of Table 4 appears below.

In the original publication, there was a mistake in Table 9, in the reporting of 18:1 (n-9) and 18:1(n-7) in the visceral fat. The corrected version of Table 9 appears below.

## 4. Error in Supplementary Materials

There was an error in the original Supplementary Material. The identification of 22:3(n-3) in the Supplementary Materials, Tables S1,S3–S5, has been corrected to 22:4(n-6). The identification of 22:4(n-6) in the Supplementary Materials, Tables S1–S5, has been corrected to 22:5(n-6).

The fatty acid composition of visceral fat has been corrected in Supplemental Table S5.

This correction was approved by the Academic Editor. The original publication has also been updated.

## Figures and Tables

**Table 4:** Total lipid (mg/100 mg) of rat organ tissues at the end of the experiment.

	Groups ^1^	
Organs	(n-3) Deficient	(n-3) Adequate
Liver TAG	1.96 ± 0.77	1.59 ± 0.39
Liver PL	3.24 ± 0.13	3.42 ± 0.36
Brain	3.82 ± 0.23	3.91 ± 0.15
Eye	0.61 ± 0.08	0.56 ± 0.05
Testis	1.56 ± 0.31	1.48 ± 0.28
Heart	2.07 ± 0.65	1.97 ± 0.23
Lung	3.56 ± 1.33	3.05 ± 0.89
Visceral fat	24.0 ± 5.7	22.8 ± 5.0

^1^ The (n-3)-deficient and (n-3)-adequate groups received peanut-oil-based (n-3) FA-deficient diet and soybean oil- based AIN-93G standard diet, respectively, for 33 days. Values for total lipid are mean ± SD, *n* = 12 for (n-3)-adequate group and *n* = 11 for (n-3)-deficient group.

**Table 9:** Fatty acid composition of the rat visceral fat, heart, and lung (% of total fatty acids) of (n-3)-deficient and (n-3)-adequate groups.

Fatty Acids	Groups ^1^					
	Visceral Fat		Heart		Lung	
	(n-3) Deficient	(n-3) Adequate	(n-3) Deficient	(n-3) Adequate	(n-3) Deficient	(n-3) Adequate
18:1(n-9), OA	44.99 ± 1.1 ^a^	31.21 ± 1.17 ^b^	14.78 ± 8.71 ^a^	7.79 ± 2.61 ^b^	28.49 ± 6.34 ^a^	20.14 ± 4.09 ^b^
18:2(n-6), LA	20.64 ± 1.06 ^a^	30.16 ± 2.48 ^b^	18.12 ± 2 ^a^	23.29 ± 1.84 ^b^	12 ± 2.41 ^a^	15.8 ± 3.63 ^b^
18:3(n-3), ALA	0.36 ± 0.05 ^a^	2.52 ± 0.19 ^b^	0.08 ± 0.05 ^a^	0.41 ± 0.18 ^b^	0.16 ± 0.05 ^a^	0.89 ± 0.26 ^b^
20:4(n-6), ARA	0.27 ± 0.04 ^a^	0.39 ± 0.07 ^b^	19.94 ± 6.65	19.68 ± 2.56	7.88 ± 3.35	8.62 ± 2.7
20:5(n-3), EPA	0.01 ± 0.01 ^a^	0.02 ± 0.01 ^b^	0.02 ± 0.01 ^a^	0.08 ± 0.03 ^b^	0.02 ± 0.01 ^a^	0.14 ± 0.05 ^b^
22:5(n-6), DPAn-6	0.03 ± 0.01	0.03 ± 0.01	2.44 ± 1.19 ^a^	0.8 ± 0.22 ^b^	0.48 ± 0.23 ^a^	0.2 ± 0.08 ^b^
22:5(n-3), DPA	nd^2^	nd^2^	0.27 ± 0.11 ^a^	0.89 ± 0.23 ^b^	0.07 ± 0.04 ^a^	0.37 ± 0.15 ^b^
22:6(n-3), DHA	0.01 ± 0.01 ^a^	0.09 ± 0.02 ^b^	2.97 ± 1.12 ^a^	6.91 ± 1.22 ^b^	0.36 ± 0.13 ^a^	0.92 ± 0.29 ^b^
Total (n-6)	21.3 ± 1.1 ^a^	31.0 ± 2.5 ^b^	41.13 ± 9.09	44.55 ± 2.67	21.09 ± 1.99 ^a^	25.61 ± 2.53 ^b^
Total (n-3)	0.5 ± 0.1 ^a^	2.8 ± 0.3 ^b^	4.55 ± 1.44 ^a^	9.21 ± 1.36 ^b^	2.30 ± 0.87 ^a^	3.97 ± 0.8 ^b^
Total PUFA	21.7 ± 1.1 ^a^	33.7 ± 2.6 ^b^	45.7 ± 10.46 ^a^	53.75 ± 3.24 ^b^	23.39 ± 2.76 ^a^	29.57 ± 2.83 ^b^

^1^ The (n-3)-deficient and (n-3)-adequate groups received peanut-oil-based (n-3) FA-deficient diet and soybean oil- based AIN-93G standard diet, respectively, for 33 days. Values are mean (relative % of total FAs) ± SD, *n* = 12 for (n-3)-adequate group and *n* = 11 for (n-3)-deficient group. Values with different superscripts indicate significant differences between the (n-3)-deficient and (n-3)-adequate feedings (*p* < 0.05). PUFA, polyunsaturated fatty acids; ^2^ nd, not detected.
