# Supplementary material for: Tissue-Specific Content of Polyunsaturated Fatty Acids in (n-3) Deficiency State of Rats
Source: Foods. 2022 Jan 12;11(2):208. doi: 10.3390/foods11020208 (PMC8774705; doi:10.3390/foods11020208)
Supplement: Supplementary file 1 [file foods-11-00208-s001.zip › foods-1515668-supplementary.pdf]

SUPPLEMENTAL INFORMATION

Tissue specific content of polyunsaturated fatty acids in (n-3) deficiency state of rats

Amruta Kulkarni[a], Ai Zhao[b], Baoru Yang[a], Yumei Zhang[c, *], Kaisa M. Linderborg[a, *]

[a] Food Chemistry and Food Development, Department of Life Technologies, University of Turku, Finland

[b] Vanke school of public health, Tsinghua University, Beijing, China

[c] Department of Nutrition & Food Hygiene, School of Public Health, Peking University Health Science Center, Beijing, China

* Corresponding authors

Supplemental Figure S1: Animal trial model diagram showing the feeding of two groups and housing system during the experiment.

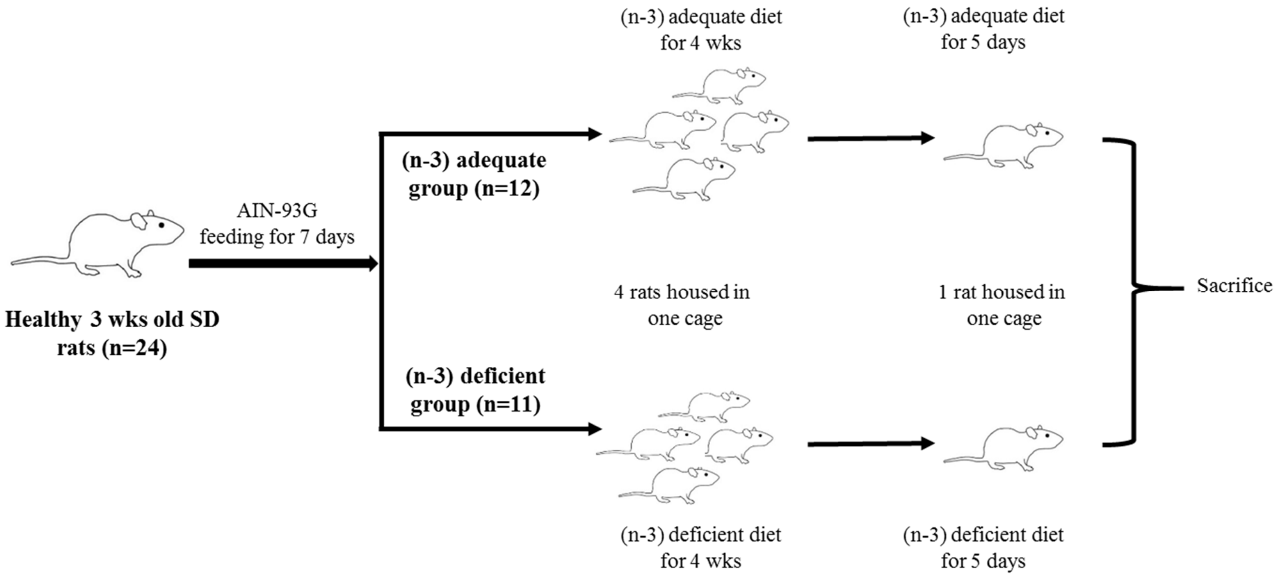

Supplemental Table S1: Glycerol-bound fatty acids and total (free and esterified) fatty acids (µg/100 mg) excreted in the rat feces collected during the last 5 days of the experiment.

Fatty acids	Groups[1]			
	(n-3) deficient	(n-3) adequate	(n-3) deficient	(n-3) adequate
	Glycerol-bound fatty acids		Total fatty acids	
12:0	0.56±0.14	0.54±0.11	2.21±0.72 [a]	2.83±0.63 [b]
14:0	4.32±0.63	3.86±0.53	10.57±2.21 [a]	12.44±1.68 [b]
14:1(n-5)	0.77±0.22	0.78±0.19	0.17±0.07	0.24±0.09
16:0	43.12±5.74 [a]	37.04±4.87 [b]	365.16±100.2	388.19±80.11
16:1(n-7)	0.53±0.09	0.52±0.14	2.21±0.38	2.07±0.3
18:0	15.22±6.98	19.74±12.77	311.71±75.3	301.73±70.8
18:1(n-9), OA	18.84±4.51 [a]	12.66±1.76 [b]	186.96±99.24 [a]	83.63±17.57 [b]
18:1(n-7)	3.5±0.57	3.04±0.52	12.95±3.79	14.34±2.8
18:2(n-6), LA	6.31±0.92 [a]	7.56±1.25 [b]	58.28±28.81	63.99±16.89
18:3(n-6)	0.24±0.03	0.23±0.04	2.63±0.27	2.69±0.71
18:3(n-3), ALA	0.2±0.1 [a]	0.42±0.06 [b]	1.14±0.84 [a]	4.64±1.31 [b]
20:0	1.71±0.6 [a]	1.09±0.15 [b]	113.34±20.28 [a]	35.73±5.45 [b]
20:1(n-9)	1.16±0.34 [a]	0.34±0.04 [b]	16.93±8.99 [a]	2.7±0.43 [b]
20:2(n-6)	0.24±0.05	0.26±0.06	1.72±0.85	2.17±0.6
20:3(n-6)	0.5±0.15	0.39±0.17	2.96±1.88	2±1.65
20:4(n-6), ARA	1.96±0.29	1.72±0.53	7.13±1.2 [a]	5.18±1.24 [b]
20:3(n-3)	0.41±0.16	0.49±0.23	1.33±0.57	1.01±0.45
20:5(n-3), EPA	0.16±0.08	0.09±0.05	0.09±0.05 [a]	0.16±0.07 [b]
22:0	3.53±1.24 [a]	1.94±0.27 [b]	270.11±40.14 [a]	54.1±7.04 [b]
22:1(n-9)	0.41±0.2 [a]	0.7±0.25 [b]	7.48±1.76 [a]	3.22±0.83 [b]
22:2(n-6)	0.28±0.12	0.32±0.09	5.56±1.79 [a]	4.11±1.33 [b]
22:3(n-3)	0.24±0.06 [a]	0.17±0.07 [b]	1.54±0.38 [a]	0.85±0.22 [b]
22:4(n-6), DTA	0.71±0.3	0.93±0.46	1.31±0.84 [a]	0.44±0.15 [b]
22:5(n-3)	0.02±0.02	0.01±0	0.21±0.1	0.17±0.09
24:0	3.92±0.99	3.74±0.69	187.17±22.2 [a]	50.95±5.27 [b]
22:6(n-3), DHA	0.13±0.05	0.17±0.04	0.62±0.21	0.77±0.26
24:1(n-9)	0.12±0.05	0.11±0.06	2.47±0.99 [a]	1.43±0.36 [b]
**Total SFA**	72.42±12.21	67.97±15.7	1260.29±232.35 [a]	846.01±164.21 [b]
**Total MUFA**	25.36±4.84 [a]	18.17±2.47 [b]	229.2±114.09 [a]	107.66±20.47 [b]
**Total (n-6)**	10.25±1.27	11.44±1.93	79.64±30.66	80.62±18.93
**Total (n-3)**	1.19±0.2	1.38±0.28	4.96±1.45 [a]	7.62±1.56 [b]
**Total PUFA**	11.45±1.24	12.83±2.17	84.6±31.79	88.24±20.42
TL (µg/100 mg)	141.6±16.9	127.5±21.5	1635.0±364.0 [a]	1098.0±196.9 [b]

[1] The (n-3) deficient and (n-3) adequate groups received peanut oil based (n-3) FA deficient diet and soy bean oil based AIN-93G standard diet respectively for 33 days.

Values are mean (µg/100 mg) ± SD, n = 12 for (n-3) adequate group and n = 11 for (n-3) deficient group. Values with different superscripts indicate significant differences between the (n-3) deficient and (n-3) adequate feedings ($p < 0.05$).

SFA: saturated fatty acids; MUFA: monounsaturated fatty acids; PUFA: polyunsaturated fatty acids; TL: Total lipids.

Supplemental Table S2: Fatty acids of the TAG and PL fractions of fasting rat plasma (% of total fatty acids) of (n-3) deficient and (n-3) adequate groups.

Fatty acids	Groups[1]			
	(n-3) deficient	(n-3) adequate	(n-3) deficient	(n-3) adequate
	TAG		PL	
14:0	1.36±1.19	1.2±1.03	0.2±0.03 [a]	0.25±0.03 [b]
16:0	20.7±1.53 [a]	18.29±1.43 [b]	23.06±0.87 [a]	24.22±0.74 [b]
16:1(n-7)	1.69±0.42 [a]	2.35±0.54 [b]	0.37±0.09 [a]	0.51±0.06 [b]
18:0	5.6±2.35	5.53±1.47	23.06±1.46 [a]	21.05±1.45 [b]
18:1(n-9), OA	28.32±3.3 [a]	19.81±2.3 [b]	5.18±0.5 [a]	3.66±0.3 [b]
18:1(n-7)	2.27±0.34	2.5±0.37	2.41±0.31 [a]	3.26±0.47 [b]
18:2(n-6), LA	22.68±1.98 [a]	28.24±2.87 [b]	10.72±1.56 [a]	12.97±1.52 [b]
18:3(n-6)	0.52±0.14	0.58±0.14	0.03±0.01 [a]	0.05±0.01 [b]
18:3(n-3), ALA	0.5±0.17 [a]	2.19±0.35 [b]	0.02±0.01 [a]	0.04±0 [b]
20:0	0.33±0.09	0.42±0.22	0.17±0.08	0.14±0.12
20:1(n-9)	0.85±0.48	0.74±0.42	0.23±0.04 [a]	0.15±0.03 [b]
20:2(n-6)	0.79±0.05 [a]	0.54±0.11 [b]	0.49±0.15	0.59±0.13
20:3(n-6)	nd[2]	nd[2]	0.5±0.11 [a]	0.79±0.12 [b]
20:4(n-6), ARA	11.59±2.64	12.39±4.96	27.72±1.69 [a]	24.93±0.91 [b]
20:5(n-3), EPA	0.25±0.29 [a]	1.24±0.3 [b]	0.01±0 [a]	0.05±0 [b]
22:0	0.2±0.08 [a]	0.03±0.01 [b]	0.04±0.01 [a]	0.02±0 [b]
22:1(n-9)	0.24±0.12	0.39±0.32	0.28±0.11	0.23±0.06
22:4(n-6), DTA	0.99±0.32 [a]	0.33±0.19 [b]	1.66±0.4 [a]	0.32±0.15 [b]
22:5(n-3)	0.12±0.05	0.09±0.1	0.02±0.01	0.01±0
24:0	0.17±0.11 [a]	0.47±0.19 [b]	0.27±0.05 [a]	0.62±0.11 [b]
22:6(n-3), DHA	0.73±0.15 [a]	2.58±0.66 [b]	3.2±0.44 [a]	5.74±0.97 [b]
**Total SFA**	**28.38±2.98**	**25.96±1.63**	**46.83±0.99**	**46.34±1.01**
**Total MUFA**	**33.39±3.54 [a]**	**25.81±2.87 [b]**	**8.49±0.77 [a]**	**7.83±0.6 [b]**
**Total (n-6) FA**	**36.59±2.17 [a]**	**42.1±3.67 [b]**	**41.14±0.66 [a]**	**39.67±1.1 [b]**
**Total (n-3) FA**	**1.61±0.36 [a]**	**6.1±0.56 [b]**	**3.27±0.43 [a]**	**5.87±0.96 [b]**
**Total PUFA**	**38.21±2.35 [a]**	**48.21±3.74 [b]**	**44.41±0.75 [a]**	**45.54±0.68 [b]**
TL (µg/100 mg)	54.8±25.1	40.5±16.5	127.7±16.5	125.7±21.0

[1] The (n-3) deficient and (n-3) adequate groups received peanut oil based (n-3) FA deficient diet and soy bean oil based AIN-93G standard diet respectively for 33 days.
Values are mean (relative % of total FAs) ± SD, n = 12 for (n-3) adequate group and n = 11 for (n-3) deficient group. Values with different superscripts indicate significant differences between the (n-3) deficient and (n-3) adequate feedings ($p < 0.05$).
SFA: saturated fatty acids; MUFA: monounsaturated fatty acids; PUFA: polyunsaturated fatty acids; TL: Total lipids.
nd[2]: not detected.

Supplemental Table S3: Fatty acids of the TAG and PL fractions of rat liver (% of total fatty acids) of (n-3) deficient and (n-3) adequate groups.

Fatty acids	Groups[1]			
	(n-3) deficient	(n-3) adequate	(n-3) deficient	(n-3) adequate
	TAG		PL	
12:0	0.08±0.02	0.09±0.02	0.03±0.02	0.03±0.02
14:0	0.82±0.11	0.87±0.18	0.16±0.04	0.2±0.06
14:1(n-5)	0.1±0.03	0.12±0.03	0.05±0.05	0.07±0.11
16:0	23.65±1.96	24.01±3.01	15.05±0.48 [a]	16.28±0.75 [b]
16:1(n-7)	2.97±0.8	3.68±1.15	0.67±0.13	0.75±0.12
18:0	4.08±2.41	3.56±1.83	21.76±1 [a]	19.89±1.22 [b]
18:1(n-9), OA	34.24±4.42 [a]	24.18±2.33 [b]	4.5±0.41 [a]	3.42±0.36 [b]
18:1(n-7)	3.22±0.5 [a]	3.72±0.51 [b]	2.66±0.31 [a]	3.33±0.56 [b]
18:2(n-6), LA	19.76±2.41 [a]	26.96±6.28 [b]	9.68±1.11 [a]	11.65±1.79 [b]
18:3(n-6)	0.06±0.01 [a]	0.26±0.07 [b]	0.03±0.01 [a]	0.06±0.02 [b]
18:3(n-3), ALA	0.27±0.06 [a]	1.7±0.49 [b]	0.03±0.02 [a]	0.11±0.04 [b]
20:0	0.06±0.02 [a]	0.05±0.02 [b]	0.08±0.02 [a]	0.05±0.01 [b]
20:1(n-9)	0.17±0.07 [a]	0.12±0.03 [b]	0.15±0.06	0.12±0.03
20:2(n-6)	0.36±0.1 [a]	0.29±0.04 [b]	0.38±0.06 [a]	0.46±0.05 [b]
20:3(n-6)	0.16±0.07 [a]	0.24±0.07 [b]	0.61±0.1	0.83±0.1
20:4(n-6), ARA	7.22±4.01	6.29±2.94	35.05±0.68 [a]	31.51±0.91 [b]
20:3(n-3)	0.06±0.03	0.08±0.04	0.08±0.02	0.07±0.02
20:5(n-3), EPA	0.06±0.02 [a]	0.38±0.13 [b]	0.02±0.01 [a]	0.13±0.03 [b]
22:0	0.02±0.01	0.02±0.02	0.02±0.01 [a]	0.01±0.01 [b]
22:1(n-9)	0.04±0.04	0.06±0.07	0.01±0.01	0.01±0.01
22:2(n-6)	0.03±0.02 [a]	0.01±0.01 [b]	0.04±0.01 [a]	0.02±0.01 [b]
22:3(n-3)	0.43±0.2 [a]	0.28±0.06 [b]	0.65±0.09 [a]	0.32±0.04 [b]
22:4(n-6), DTA	0.65±0.37 [a]	0.2±0.09 [b]	2.4±0.61 [a]	0.45±0.23 [b]
22:5(n-3)	0.02±0.02 [a]	0.03±0.02 [b]	0.08±0.03	0.1±0.05
24:0	0.1±0.05 [a]	0.31±0.07 [b]	0.34±0.07 [a]	0.69±0.13 [b]
22:6(n-3), DHA	0.66±0.56 [a]	1.66±0.8 [b]	4.86±0.55 [a]	8.92±1.88 [b]
24:1(n-9)	0.02±0.03	0.01±0.01	0.01±0.01	0.01±0.01
**Total SFA**	**28.78±2.32**	**28.88±3.46**	**37.4±0.67**	**37.12±0.84**
**Total MUFA**	**40.74±5.39 [a]**	**31.87±3.47 [b]**	**8.01±0.63**	**7.68±0.67**
**Total (n-6)**	**28.21±3.86 [a]**	**34.22±5.64 [b]**	**48.15±0.63 [a]**	**44.95±2.08 [b]**
**Total (n-3)**	**1.47±0.69 [a]**	**4.11±0.74 [b]**	**5.71±0.58 [a]**	**9.62±1.86 [b]**
**Total PUFA**	**29.68±4.42 [a]**	**38.32±6.04 [b]**	**53.85±0.66 [a]**	**54.56±0.72 [b]**

[1] The (n-3) deficient and (n-3) adequate groups received peanut oil based (n-3) FA deficient diet and soy bean oil based AIN-93G standard diet respectively for 33 days.

Values are mean (relative % of total FAs) ± SD, n = 12 for (n-3) adequate group and n = 11 for (n-3) deficient group. Values with different superscripts indicate significant differences between the (n-3) deficient and (n-3) adequate feedings ($p < 0.05$).

SFA: saturated fatty acids; MUFA: monounsaturated fatty acids; PUFA: polyunsaturated fatty acids.

Supplemental Table S4: Fatty acid composition of the rat brain, eye, and testis (% of total fatty acids) of (n-3) deficient and (n-3) adequate groups.

Fatty acids	Groups[1]					
	Brain		Eye		Testis	
	(n-3) deficient	(n-3) adequate	(n-3) deficient	(n-3) adequate	(n-3) deficient	(n-3) adequate
12:0	0.14±0.09	0.12±0.03	0.2±0.07	0.17±0.06	0.17±0.05	0.16±0.04
14:0	0.24±0.04	0.27±0.06	1.34±0.16 [a]	1.21±0.1 [b]	0.89±0.24	0.88±0.23
14:1(n-5)	nd[2]	nd[2]	0.12±0.07	0.11±0.04	0.13±0.05	0.12±0.04
16:0	18.28±1.16	18.65±0.88	20.97±0.6	20.86±0.62	31.7±2.01	32.31±1.89
16:1(n-7)	0.3±0.03	0.32±0.03	2.89±0.86	2.6±0.6	2.32±1.15	1.96±0.71
18:0	18.51±0.49	18.48±0.63	14.93±1.76 [a]	16.6±1.62 [b]	6.22±0.75	6.42±0.48
18:1(n-9), OA	14.85±0.85	14.49±0.75	15.85±2.69 [a]	13.21±1.62 [b]	17.25±3.89 [a]	12.48±2.08 [b]
18:1(n-7)	3.17±0.2	3.04±0.19	3.01±0.15	3.14±0.22	2.37±0.22	2.4±0.19
18:2(n-6), LA	0.61±0.08 [a]	0.74±0.09 [b]	5.28±1.94	5.75±1.86	7.77±1.97	9.96±3.08
18:3(n-6)	0.02±0.02	0.02±0.01	0.12±0.02	0.12±0.01	0.24±0.17	0.13±0.12
18:3(n-3), ALA	0.02±0.01	0.02±0.01	0.13±0.24	0.24±0.12	0.14±0.07 [a]	0.62±0.32 [b]
20:0	0.59±0.17	0.54±0.12	0.31±0.03	0.33±0.06	0.15±0.02	0.13±0.03
20:1(n-9)	0.45±0.12	0.39±0.1	0.23±0.06	0.18±0.01	0.2±0.05 [a]	0.11±0.02 [b]
20:2(n-6)	0.24±0.06	0.24±0.05	0.34±0.02 [a]	0.37±0.03 [b]	0.25±0.03	0.26±0.04
20:3(n-6)	0.39±0.03 [a]	0.43±0.05 [b]	0.27±0.03 [a]	0.33±0.04 [b]	0.83±0.13 [a]	1.04±0.12 [b]
20:4(n-6), ARA	10.39±1.84	10.86±0.6	11.72±0.95	11.5±0.57	14.45±2.49	15.24±2.35
20:3(n-3)	nd[2]	nd[2]	0.02±0.03	0.02±0.02	0.1±0.02	0.11±0.02
20:5(n-3), EPA	nd [2]	nd[2]	0.02±0.01 [a]	0.1±0.1 [b]	0.01±0.01 [a]	0.04±0.02 [b]
22:0	1.02±1.38	0.55±0.12	0.3±0.03	0.31±0.09	0.13±0.02	0.24±0.45
22:1(n-9)	0.22±0.1	0.18±0.04	0.11±0.03	0.1±0.04	0.7±0.12	0.7±0.14
22:2(n-6)	0.04±0.03	0.03±0.01	0.05±0.02	0.04±0.02	0.04±0.01	0.05±0.06
22:3(n-3)	2.95±0.5	2.83±0.15	1.88±0.15 [a]	1.64±0.12 [b]	1.65±0.28	1.71±0.23
22:4(n-6), DTA	1.45±0.3 [a]	0.73±0.34 [b]	1.51±0.34 [a]	0.54±0.1 [b]	11.26±1.89	11.55±1.76
22:5(n-3)	nd[2]	nd[2]	nd[2]	nd[2]	nd[2]	nd[2]

24:0	1.07±0.27	0.97±0.16	0.61±0.16 [a]	1.01±0.11 [b]	0.09±0.02 [a]	0.08±0.02 [b]
22:6(n-3), DHA	13.59±1.93 [a]	15.8±1.04 [b]	16.9±2.42	18.71±2.55	0.58±0.14 [a]	0.94±0.21 [b]
24:1(n-9)	2.03±1.74	1.36±0.32	0.41±0.03	0.42±0.03	0.05±0.02	0.05±0.01
**Total SFA**	**39.83±1.57**	**39.57±0.98**	**38.63±2.15** [a]	**40.45±1.88** [b]	**39.32±2.4**	**40.18±1.84**
**Total MUFA**	**20.98±2.56**	**19.76±1.3**	**22.6±3.31** [a]	**19.73±2.25** [b]	**22.99±5.14** [a]	**17.78±2.81** [b]
**Total (n-6)**	**13.11±1.74**	**13.01±0.7**	**19.28±1.45**	**18.63±1.88**	**34.8±2.56** [a]	**38.2±1.08** [b]
**Total (n-3)**	**16.55±2.39** [a]	**18.64±1.01** [b]	**18.93±2.48**	**20.7±2.46**	**2.46±0.35** [a]	**3.39±0.16** [b]
**Total PUFA**	**29.66±4.08**	**31.65±1.33**	**38.21±2.04**	**39.32±1.39**	**37.26±2.87** [a]	**41.59±1.14** [b]

[1] The (n-3) deficient and (n-3) adequate groups received peanut oil based (n-3) FA deficient diet and soy bean oil based AIN-93G standard diet respectively for 33 days.
Values are mean (relative % of total FAs) ± SD, n = 12 for (n-3) adequate group and n = 11 for (n-3) deficient group. Values with different superscripts indicate significant differences between the (n-3) deficient and (n-3) adequate feedings ($p < 0.05$).
SFA: saturated fatty acids; MUFA: monounsaturated fatty acids; PUFA: polyunsaturated fatty acids.
nd [2]: not detected.

Supplemental Table S5: Fatty acid composition of the rat visceral fat, heart, and lung (% of total fatty acids) of (n-3) deficient and (n-3) adequate groups.

Fatty acids	Groups[1]					
	Visceral fat		Heart		Lung	
	(n-3) deficient	(n-3) adequate	(n-3) deficient	(n-3) adequate	(n-3) deficient	(n-3) adequate
12:0	0.3±0.03 [a]	0.28±0.04 [b]	0.32±0.06 [a]	0.26±0.07 [b]	0.39±0.18	0.42±0.12
14:0	1.62±0.32 [a]	1.9±0.13 [b]	0.94±0.62	0.65±0.22	2.06±0.26	2.2±0.22
14:1(n-5)	0.17±0.06 [a]	0.24±0.05 [b]	0.1±0.09	0.08±0.07	0.35±0.15	0.29±0.11
16:0	20.75±1.57	21.13±1.44	14.48±3.74	13.45±1.17	27.06±2.4	28.45±2.19
16:1(n-7)	4.92±0.75 [a]	6.09±0.85 [b]	1.69±1.79	1.22±0.73	3.67±1.56	4.15±1.44
18:0	2±0.47	2.37±0.51	16.71±4.24	17.64±1.42	8.86±3.19	9.1±1.8
18:1(n-9), OA	10.35±4.1	11.01±3.37	14.78±8.71 [a]	7.79±2.61 [b]	28.49±6.34 [a]	20.14±4.09 [b]
18:1(n-7)	36.59±3.97 [a]	21.86±4.04 [b]	3.5±0.38 [a]	3.84±0.35 [b]	2.47±0.33 [a]	2.75±0.29 [b]
18:2(n-6), LA	20.85±1.04 [a]	30.92±2.53 [b]	18.12±2 [a]	23.29±1.84 [b]	12±2.41 [a]	15.8±3.63 [b]
18:3(n-6)	0.1±0.02 [a]	0.11±0.02 [b]	0.02±0.01 [a]	0.09±0.03 [b]	0.03±0.01 [a]	0.12±0.04 [b]
18:3(n-3), ALA	0.37±0.06 [a]	2.59±0.21 [b]	0.08±0.05 [a]	0.41±0.18 [b]	0.16±0.05 [a]	0.89±0.26 [b]
20:0	0.34±0.07 [a]	0.13±0.03 [b]	0.39±0.1 [a]	0.27±0.02 [b]	0.42±0.09	0.36±0.1
20:1(n-9)	0.58±0.05 [a]	0.22±0.02 [b]	0.26±0.08 [a]	0.1±0.02 [b]	0.48±0.04 [a]	0.23±0.03 [b]
20:2(n-6)	0.14±0.03 [a]	0.2±0.03 [b]	0.28±0.06	0.28±0.03	0.31±0.09	0.37±0.08
20:3(n-6)	0.03±0.01 [a]	0.08±0.02 [b]	0.31±0.09 [a]	0.41±0.08 [b]	0.33±0.14 [a]	0.48±0.14 [b]
20:4(n-6), ARA	0.29±0.05 [a]	0.41±0.08 [b]	19.94±6.65	19.68±2.56	7.88±3.35	8.62±2.7
20:3(n-3)	0.01±0.01 [a]	0.04±0.01 [b]	0.08±0.03	0.09±0.02	0.04±0.03 [a]	0.07±0.03 [b]
20:5(n-3), EPA	0.01±0.01 [a]	0.03±0.01 [b]	0.02±0.01 [a]	0.08±0.03 [b]	0.02±0.01 [a]	0.14±0.05 [b]
22:0	0.21±0.05 [a]	0.06±0.02 [b]	0.47±0.11 [a]	0.29±0.04 [b]	0.52±0.15	0.53±0.17
22:1(n-9)	0.03±0.01 [a]	0.02±0.01 [b]	0.07±0.05	0.08±0.12	0.22±0.08	0.18±0.05
22:2(n-6)	0.01±0.01 [a]	0.01±0.01 [b]	0.05±0.02 [a]	0.03±0.01 [b]	0.1±0.05 [a]	0.05±0.02 [b]
22:3(n-3)	0.05±0.02 [a]	0.07±0.02 [b]	1.14±0.27 [a]	0.86±0.14 [b]	1.68±0.73	1.6±0.57
22:4(n-6), DTA	0.04±0.02	0.04±0.02	2.44±1.19 [a]	0.8±0.22 [b]	0.48±0.23 [a]	0.2±0.08 [b]
22:5(n-3)	nd[2]	nd[2]	0.27±0.11 [a]	0.89±0.23 [b]	0.07±0.04 [a]	0.37±0.15 [b]

24:0	0.11±0.03 [a]	0.02±0.01 [b]	0.16±0.03 [a]	0.09±0.02 [b]	0.46±0.17	0.44±0.14
22:6(n-3), DHA	0.02±0.01 [a]	0.1±0.03 [b]	2.97±1.12 [a]	6.91±1.22 [b]	0.36±0.13 [a]	0.92±0.29 [b]
24:1(n-9)	0.03±0.01	0.03±0.01	0.06±0.07	0.03±0.01	0.61±0.29	0.58±0.19
**Total SFA**	**25.3±1.66**	**25.86±1.85**	**33.46±1.33**	**32.62±0.83**	**39.75±5.43**	**41.48±4.15**
**Total MUFA**	**52.65±1.17 [a]**	**39.43±2.07 [b]**	**20.44±10.47 [a]**	**13.12±3.3 [b]**	**36.26±7.58 [a]**	**28.29±5.26 [b]**
**Total (n-6)**	**21.43±1.03 [a]**	**31.74±2.48 [b]**	**41.13±9.09**	**44.55±2.67**	**21.09±1.99 [a]**	**25.61±2.53 [b]**
**Total (n-3)**	**0.44±0.06 [a]**	**2.81±0.22 [b]**	**4.55±1.44 [a]**	**9.21±1.36 [b]**	**2.3±0.87 [a]**	**3.97±0.8 [b]**
**Total PUFA**	**21.87±1.02 [a]**	**34.55±2.64 [b]**	**45.68±10.46 [a]**	**53.75±3.24 [b]**	**23.39±2.76 [a]**	**29.57±2.83 [b]**

[1] The (n-3) deficient and (n-3) adequate groups received peanut oil based (n-3) FA deficient diet and soy bean oil based AIN-93G standard diet respectively for 33 days.

Values are mean (relative % of total FAs) ± SD, n = 12 for (n-3) adequate group and n = 11 for (n-3) deficient group. Values with different superscripts indicate significant differences between the (n-3) deficient and (n-3) adequate feedings (p < 0.05).

SFA: saturated fatty acids.

MUFA: monounsaturated fatty acids.

PUFA: polyunsaturated fatty acids.

nd [2]: not detected.
